# Bioprospection of rattlesnake venom peptide fractions with anti-adipose and anti-insulin resistance activity *in vitro*

**DOI:** 10.1016/j.toxcx.2024.100209

**Published:** 2024-09-19

**Authors:** David Meléndez-Martínez, Erika Ortega-Hernández, Edwin Estefan Reza-Zaldívar, Alejandro Carbajal-Saucedo, Gustavo Arnaud-Franco, Ana Gatica-Colima, Luis Fernando Plenge-Tellechea, Marilena Antunes-Ricardo, Daniel A. Jacobo-Velázquez, Karla Mayolo-Deloisa, Omar Lozano, Marco Rito-Palomares, Jorge Benavides

**Affiliations:** a Tecnologico de Monterrey, Institute for Obesity Research, Ave. Eugenio Garza Sada Sur 2501, C.P. 64849, Monterrey, N.L., Mexico; b Tecnologico de Monterrey, Escuela de Ingeniería y Ciencias, Centro de Biotecnología-FEMSA, Ave. Eugenio Garza Sada Sur 2501, C.P. 64849, Monterrey, N.L., Mexico; c Tecnologico de Monterrey, Escuela de Ingeniería y Ciencias, Ave. General Ramón Corona 2514, Zapopan, 45201, Mexico; d Tecnologico de Monterrey, Institute for Obesity Research, Ave. General Ramón Corona 2514, Zapopan, 45201, Mexico; e Universidad Autónoma de Nuevo León, Facultad de Ciencias Biológicas, Laboratorio de Herpetología, San Nicolás de los Garza, Nuevo León, C.P. 66450, Mexico; f Centro de Investigaciones Biológicas del Noroeste, S.C. Instituto Politécnico Nacional, 195, Col. Playa Palo de Santa Rita, La Paz, B.C.S, 23090, Mexico; g Departamento de Ciencias Químico-Biológicas, Instituto de Ciencias Biomédicas, Universidad Autónoma de Ciudad Juárez, Anillo Envolvente del PRONAF y Estocolmo s/n, Ciudad Juárez, Chih, 32310, Mexico; h Tecnologico de Monterrey, Escuela de Medicina y Ciencias de la Salud, Ave. Morones Prieto 3000 Pte, C.P. 64460, Monterrey, N.L., Mexico

**Keywords:** Adipocyte lipid accumulation, Bioactive peptides, *Crotalus*, Insulin resistance, Venom, Crotamine-like peptides

## Abstract

Animal venoms are natural products that have served as a source of novel molecules that have inspired novel drugs for several diseases, including for metabolic diseases such as type-2 diabetes and obesity. From venoms, toxins such as exendin-4 (*Heloderma suspectum*) and crotamine (*Crotalus durissus terrificus*) have demonstrated their potential as treatments for obesity. Moreover, other toxins such as Phospholipases A_2_ and Disintegrins have shown their potential to modulate insulin secretion in vitro. This suggests an unexplored diversity of venom peptides with a potential anti-obesogenic in Mexican rattlesnake venoms. For that reason, this study explored the in vitro effect of Crotalus venom peptide-rich fractions on models for insulin resistance, adipocyte lipid accumulation, antioxidant activity, and inflammation process through nitric oxide production inhibition. Our results demonstrated that the peptide-rich fractions of *C. aquilus, C. ravus*, and *C. scutulatus scutulatus* were capable of reverting insulin resistance, enhancing glucose consumption to normal control; *C. culminatus, C. molossus oaxacus*, and *C. polystictus* diminished the lipid accumulation on adipocytes by 20%; *C. aquilus, C. ravus*, and *C. s. salvini* had the most significant cellular antioxidant activity, having nearly 80% of ROS inhibition. *C. aquilus, C. pyrrhus,* and *C. s. salvini* inhibited nitric oxide production by about 85%. We demonstrated the potential of these peptides from *Crotalus* venoms to develop novel drugs to treat type-2 diabetes and obesity. Moreover, we described for the first time that *Crotalus* venom peptide fractions have antioxidant and inflammatory properties in vitro models.

## Highlights

• Bioactivity of venom peptide fractions of 18 Crotalus species was characterized.
• *C. ravus* venom peptide fraction was the best reverting insulin resistance in vitro.
• *C. catalinensis* and *C. m. oaxacus* reduced lipid accumulation in vitro adipocytes.
• *C. aquilus* and *C. s. salvini* peptides prevented the antioxidant activity in vitro.
• *C. aquilus, C. pyrrhus,* and *C. s. salvini* inhibited nitric oxide production in vitro.

## Graphical abstract

**Figure:**
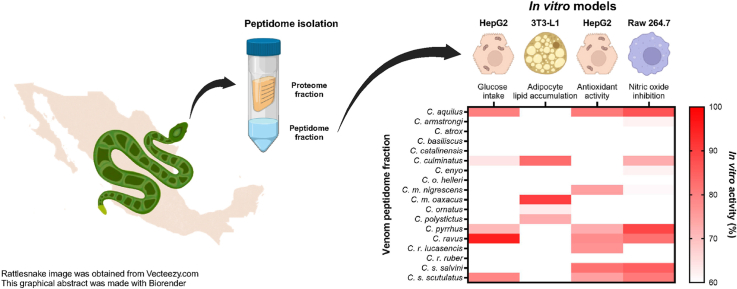

## 1 Introduction

Obesity is a chronic disease characterized by an excessive accumulation of body fat (Izquierdo-Torres et al., 2022; Lustig et al., 2022). In Mexico, overweight and obesity are health problems that affect about 73% of the adult population. Moreover, this problem affects childhood (6.6 million) and adolescence (7.3 million) (Shamah-Levy et al., 2022). Overweight and obesity are considered risk factors for non-communicable diseases such as metabolic syndrome, type 2 diabetes, insulin resistance, and cardiovascular diseases, among others (Chang and Hung, 2022; Lustig et al., 2022; Verduci et al., 2022). It can even decrease life expectancy from 5 to 20 years (Fontaine et al., 2003). Several drugs have been developed to treat obesity but have limited efficacy, and they present significant side effects such as cardiovascular and cerebrovascular problems, cancer, and psychological problems such as depression or suicidal ideation (Müller et al., 2021).

In recent years, many research groups have been focused on searching for novel molecules with less or null side effects. For that reason, natural products have been studied as a source of molecules for obesity treatment and prevention (Chang and Hung, 2022; Wen et al., 2022), including secretions from venomous animals (Amatya et al., 2020; Coulter-Parkhill et al., 2021; de Oliveira et al., 2023; Oliveira et al., 2022). Exendin-4 is a peptide isolated from Gila monster lizard (*Heloderma suspectum*) that inspired drugs such as Byetta®, Bydureon®, Exenatide® or Semaglutide® to treat type 2 diabetes and obesity (Coulter-Parkhill et al., 2021; Wen et al., 2022; Wilding et al., 2021). Another reptile toxin is crotamine (Ctm), isolated from the South American rattlesnake (*Crotalus durissus terrificus*) (Rádis-Baptista, 2021). *In vivo* experimentation on mice demonstrated that Ctm decreased body weight gain and white adipose tissue, promoting adipose browning. Also, increased glucose tolerance and diminished the lipid levels in bloodstream (Marinovic et al., 2018). Moreover, other Ctm-like peptide isolated from *C. d. cascavella* can promote insulin secretion in pancreatic β-cells during insulin resistance (IR) (Toyama et al., 2005). Furthermore, venom fractions obtained from *C. adamanteus* and *C. vegrandis* containing phospholipases A_2_ (PLA_2_) and disintegrins (Dis) were demonstrated to be insulinotropic in vitro models (Moore et al., 2015).

The information reported about the application of new toxin-inspired drugs to treat type-2 diabetes and obesity is scarce and almost limited to Exendin-4 and Ctm. Nevertheless, this information suggests that an unexplored diversity of peptides with a role in obesity control may exist in reptile venoms, particularly in the rattlesnakes (*Crotalus* spp.) from which various Ctm-like peptides have been described from other species than *C. d. terrificus* (Borja et al., 2018; Ponce-López et al., 2021; Ponce-Soto et al., 2007; Salazar et al., 2020; Toyama et al., 2005). Thus, this study explored the effect of 18 Mexican rattlesnake (*Crotalus*) venom peptide-rich fractions in obesity related in vitro models. These models include a lipid accumulation model in adipocyte-differentiated 3T3-L1 cells and insulin resistance model in HepG2 cells. Moreover, cellular antioxidant activity and anti-inflammatory activities were assayed in HepG2 and Raw 264.7 cell lines, respectively. The last two models were evaluated as obesity generates an abnormal hypertrophy in adipose tissue and disturbances in lipid and glucose metabolism, leading to chronic inflammation and oxidative stress (Morigny et al., 2021; Włodarczyk and Nowicka, 2019). These results will provide novel insights into the bioprospection of the rattlesnake venom peptide-rich fractions and their potential to explore new molecule alternatives to develop new toxin-inspired drugs to treat type-2 diabetes and obesity.

## 2 Materials and methods

### 2.1 Chemicals

The following chemicals: 2′,7′-Dichlorofluorescein diacetate (DCFH-DA), 2,2′-azobis (2-methylpropionamidine)dihydrochloride (AAPH), 3-Isobutyl-1-methylxanthine (IBMX), aluminum sulfate-(14–18)-hydrate, bovine insulin solution, dexamethasone, human insulin solution, glibenclamide (Glib), Glucose (GO) Assay Kit, lipopolysaccharide (LPS), N,N′-methylenebis acrylamide, sodium dodecyl sulfate, and sulfuric acid were obtained from Sigma Aldich (St. Louis, MO, USA). Dulbecco Modified Eagle Medium (DMEM), high glucose DMEM, fetal bovine serum, phosphate-buffered saline (PBS, pH 7.4), 0.25% trypsin-0.1% EDTA, and Penicillin-Streptomycin antibiotic (Pen-Strep) were acquired from GIBCO (Carlsbad, CA, USA). Acrylamide, Coomassie brilliant blue G-250, Precision Plus Protein™ Dual Xtra, tricine, and tris were acquired from Bio-Rad (Hercules, CA, USA). Griess Reagent System and CellTiter 96 AQ_ueous_ One Solution Cell Proliferation Assay kits were acquired from Promega (G2930, Madison, WI, USA). Bovine calf serum was acquired from Corning (Glendale, AZ, USA). Ethanol was acquired from Desarrollo de Especialidades Químicas (Monterrey, NL, México). Orthophosphoric acid was acquired from J.T. Baker (Phillipsburg, NJ, USA). Pierce Quantitative Colorimetric Peptide Assay Kit was acquired from Thermo Fisher Scientific (Waltham, MA, USA).

### *2.2* *Crotalus* venom samples

The venom samples from *Crotalus* species were obtained from specimens captured in the wild and released at the same point and from specimens kept in captivity from various private and institutional collections, listed in Table 1. Venom extraction was performed, and each sample was individually stored in liquid nitrogen, lyophilized using a Freeze Plus system (LabConco, MO, USA), and stored at −20 °C until used (Arnaud-Franco et al., 2023).

**Table 1:** *Crotalus* venom samples used for peptide-rich fraction extraction. The geographical origin of each sample that comprises the pooled venom is described.

Species (Abbreviature)	Pooled venom (mg)	ID	Municipality (Collection)
*C. aquilus* (Caq)	53.7	HK_634	Calvillo, Aguascalientes (INIRENA)
		HK_643	El Marqués, Querétaro (UAQ Vivarium)
		HK_733	Acámbaro, Michoacán (Najil Kaan Herpetarium)
*C. armstrongi* (Carm)	44.5	HK_727	Tancítaro, Michoacán (INIRENA)
		HK_728	Indapanapeo, Michoacán (INIRENA)
		HK_730	Morelia, Michoacán (INIRENA)
*C. atrox* (Catx)	60	IO_C1_Crax3	Juarez City, Chihuahua (LEBA, UACJ)
		IO_C1_Crax4	Juarez City, Chihuahua (LEBA, UACJ)
		IO_C1_Crax5	Juarez City, Chihuahua (LEBA, UACJ)
*C. basiliscus* (Cbas)	50.1	HK_447	Coquimatlan, Colima (Parque Ecologico el Palapo)
		HK_455	Coquimatlan, Colima (Parque Ecologico el Palapo)
		HK_499	Unknown (Najil Kaan Herpetarium)
*C. catalinensis* (Ccat)	46.1	HK_410	Isla Santa Catalina, Baja California Sur∗
		HK_411	Isla Santa Catalina, Baja California Sur∗
		HK_412	Isla Santa Catalina, Baja California Sur∗
*C. culminatus* (Ccul)	63.2	HK_512	Jojutla, Morelos (Najil Kaan Herpetarium)
		HK_663	Morelos (UMA El Teutle)
		HK_745	Malinalco, Estado de México (UMA Malinalcóatl)
*C. enyo* (Ceny)	46.2	IOR_C1_Ceny1	La Paz, Baja California Sur∗
		IOR_C1_Ceny2	La Paz, Baja California Sur∗
		HK_782	La Paz, Baja California Sur∗
*C. oreganus helleri* (Chell)	48.2	HK_911	Ensenada, Baja California (UABC Herpetarium)
		HK_912	Ensenada, Baja California (UABC Herpetarium)
		HK_918	Ensenada, Baja California (UABC Herpetarium)
*C. molossus nigrescens* (Cmn)	64.3	HK_339	Arteaga, Coahuila (MUDE)
		HK_353	San Juan del Río, Qerétaro∗
		HK_354	San Juan del Río, Qerétaro∗
*C. m. oaxacus* (Coax)	57.4	HK_351	Oaxaca∗
		HK_377	Amozoc, Puebla (Staku Luhua Herpetarium, UV Xalapa)
		IOR_C1_Coax1	Oaxaca∗
*C. ornatus* (Corn)	51	HK_334	Saltillo, Coahuila (MUDE)
		HK_335	Saltillo, Coahuila (MUDE)
		HK_336	Saltillo, Coahuila (MUDE)
		IO_C1_Cror8	Ascencion, Chihuahua (LEBA, UACJ)
*C. polystictus* (Cpol)	51.5	HK_393	Morelia, Michoacán (INIRENA)
		HK_394	Morelia, Michoacán (INIRENA)
		HK_638	Unknown (UAA Herpetarium)
*C. pyrrhus* (Cpyr)	44.2	HK_915	Ensenada, Baja California (UABC Herpetarium)
		HK_420	Ensenada, Baja California (UABC Herpetarium)
*C. ravus* (Crav)	49.9	HK_601	Mexico City (Facultad de ciencias Herpetarium, UNAM)
		HK_602	Mexico City (Facultad de ciencias Herpetarium, UNAM)
		HK_603	Mexico City (Facultad de ciencias Herpetarium, UNAM)
*C. ruber lucasensis* (Cluc)	56.2	IOR_C1_Cluc1	La Paz, Baja California Sur∗
		IOR_C1_Cluc2	La Paz, Baja California Sur∗
		IOR_C1_Cluc3	La Paz, Baja California Sur∗
*C. r. ruber* (Crub)	55.7	HK_794	Ensenada, Baja California (UABC Herpetarium)
		HK_795	Ensenada, Baja California (UABC Herpetarium)
		HK_917	Ensenada, Baja California (UABC Herpetarium)
*C. scutulatus salvini* (Csal)	54.3	HK_858	Perote, Veracruz∗
		HK_870	Perote, Veracruz∗
		HK_871	Tepeyahualco, Puebla∗
*C. s. scutulatus* (Cscu)	50.5	IO_C1_Crsc1	Janos, Chihuahua (LEBA, UACJ)
		IO_C1_Crsc2	Janos, Chihuahua (LEBA, UACJ)

Abbreviatures: INIRENA, Instituto de Investigaciones sobre los Recursos Naturales; LEBA, Laboratorio de Ecología y Biodiversidad Animal; MUDE, Museo del Desierto; UAA, Universidad Autónoma de Aguascalientes; UABC, Universidad Autónoma de Baja California; UACJ, Universidad Autónoma de Ciudad Juárez; UMA, Unidad de Manejo para la conservación de la Vida Silvestre; UNAM, Universidad Nacional Autónoma de México; UAQ, Universidad Autónoma de Querétaro; UV, Universidad de Veracruz.

Note: The samples with an asterisk (∗) were obtained from specimens captured in the wild and released at the same point.

### 2.3 Tricine-SDS-PAGE

The protein pattern of *Crotalus* whole venom samples was observed in Tricine-SDS-PAGE under reducing conditions according to Schägger (2006) and stained using Coomassie colloidal stain (Dyballa and Metzger, 2009). Briefly, 10 μg of each venom sample was dissolved in 5X loading buffer (250 mM Tris-HCl pH 6.8, 8% SDS, 0.1% bromophenol blue, and 40% glycerol, 10% β-mercaptoethanol), boiled and applied to a 16% Tricine-SDS-PAGE. The gels were run at 120 V until the molecular weight marker bands were completely resolved. Gels were washed trice with MilliQ water and stained overnight with Coomassie colloidal staining (0.02% CBB G-250, 5% aluminum sulfate-(14–18)-hydrate, 10% ethanol (96%), 2% orthophosphoric acid (85 %)). Then, gels were washed twice with MilliQ water, and the images were obtained with the iBright FL1500 Imaging System (Thermo Fisher Scientific, MA, USA). The apparent molecular weight of the peptide bands was calculated using Precision Plus Protein™ Dual Xtra as the molecular weight marker.

### 2.4 Peptide fractionation

*Crotalus* venom peptide fractions were isolated through ultrafiltration, as previously described by da Silva Caldeira et al. (da Silva Caldeira et al., 2021). Briefly, about 50 mg of each pooled venom was dissolved in 4 mL of H_2_O, centrifuged at 15 min for 13,500 rpm at 4 °C using a Microfuge® 22R centrifuge (Beckman Coulter, CA, USA) to remove insoluble proteins and cellular debris. The supernatants were ultrafiltered to obtain the peptide-rich fraction using a 3000 Da NMWL Amicon Ultra-4 regenerated cellulose filter. The eluate peptide-rich fraction was collected and stored at −20 °C until utilization.

### 2.5 Peptide-rich fractions quantification

*Crotalus* venom peptide-rich fractions were quantified using the Pierce Quantitative Colorimetric Peptide Assay Kit using the peptide digest as standard. Briefly, 20 μL of each peptide-rich fraction sample was mixed with 180 μL of the working reagent and incubated for 15 min at 37 °C. Absorbance was measured at 480 nm using a microplate reader (Synergy HT, Biotek, Winooski, VT, USA).

### *2.6* *In vitro* activity of peptide-rich fractions isolated from *Crotalus* snake venoms

The effects of *Crotalus* venom peptide-rich fractions were evaluated on the following in vitro models: IR, lipid accumulation, cellular antioxidant activity (CAA), and nitric oxide production inhibition.

#### 2.6.1 Cell lines

3T3-L1 Murine fibroblast cells, HepG2 human hepatocyte carcinoma cells and Raw 264.7 murine macrophage cells and were obtained from the American Collection Type Culture (ATCC®, VA, USA).

#### 2.6.2 Cell viability measurement

The viability of 3T3-L1, HepG2, and Raw 264.7 cells treated with *Crotalus* venom peptide-rich fractions was determined by the MTS [3-(4,5-dimethylthiazol-2-yl)-5-(3-carboxymethoxyphenyl)-2-(4-sulfophenyl)-2H-tetrzolium]-based CellTiter 96 AQ_ueous_ One Solution Cell Proliferation Assay. During this experiment, MTS is reduced to produce formazan by physiologically important reducing agents such as succinate dehydrogenase, NADPH and NADH. MTS reduction is mainly related to the mitochondrial electron transport, demonstrating that this measure reflects the viability of metabolically active cells (Berridge and Tan, 1993). To achieve the cell viability, different concentrations of venom peptide-rich fractions (0–20 μg/mL) were tested for 24 h in all the cell lines. After incubation, absorbance was measured at 490 nm with a 96-well microplate reader (Synergy HT, Bio-Tek, Winooski, VM, USA). Cell viability percentage (%) was calculated by dividing the absorbance of cells treated by the absorbance of the control (non-treated) cells and multiplied by 100.

#### 2.6.3 Insulin resistance

The IR model was performed according to the method described by Huang et al. (2015) with slight modifications. Human HepG2 cells were grown in DMEM supplemented with 10% FBS, and 1% Pen-Strep antibiotic and maintained at 37 °C in a humidified atmosphere of 5 % CO_2_. Cells were seeded in a 96-well plate (5 × 10^4^ cells/well) and allowed to adhere for 16–24 h. After that, the medium was replaced, and cells were incubated for 24 h with DMEM containing 25 mM D-glucose, 4 mM glutamine, and 1% FBS. Subsequently, half of the wells were treated with 50 μL insulin (5 × 10^−7^ mol/L), while the remaining wells served as controls for each sample. As a positive control for insulin resistance modulation, we used 200 mM Glib to treat the cells. Glib is a commercial drug used for the treatment of diabetes by increasing the sensitivity of peripheral tissues to insulin action.

After 24 h insulin incubation, the supplemented DMEM media was replaced with fresh media containing *Crotalus* peptide-rich fractions (5 μg/mL) for 5 h. At the end of the incubation, the amount of glucose on the medium was determined using the Glucose (GO) assay kit (Sigma-Aldrich, MO, USA) according to manufacturer's directions. The absorbance values were read at 540 nm on a Synergy HT plate reader (Bio-Tek Instruments, Inc., VT, USA). The glucose consumption was obtained by subtracting the blank wells from the treatment glucose concentrations.

#### 2.6.4 Lipid accumulation assay

The 3T3-L1 cells were grown in DMEM-high glucose supplemented with 10% bovine calf serum and 1% Pen-Strep antibiotic at 37 °C and 5% CO_2_. After reaching confluency, cells were seeded in a 24-well plate (1.5-2 × 10^4^ cells/well) and differentiated according to Zebisch et al. (2012). Confluent wells were differentiated with DMEM high glucose supplemented with 10% FBS, 0.5 mM IBMX, 0.25 μM dexamethasone, and 5 μg/mL insulin. From day forth to sixth, the cells were cultured with DMEM high glucose supplemented with 10% FBS and 5 μg/mL insulin. From the seventh to the ninth day, the cells were cultured with DMEM high glucose supplemented only with 10% FBS. All venom peptide-rich fractions dissolved in PBS were added in every cell media change, the effect of PBS alone was assayed (defined as vehicle in Fig. 3). The 3T3-L1 cells were considered fully differentiated on the ninth day of culture.

The lipid accumulation in 3T3-L1 adipocytes was quantified using Oil Red O staining. Once the differentiation was finished, the media was discarded, and the cells were washed twice with 0.01 M PBS pH 7.4. After, the cells were fixed with 4% paraformaldehyde for 15 min and washed twice with PBS. Then, the cells were permeated with 60% isopropanol for 15 s. The cells were stained with Oil Red O solution (5 mg/L in isopropanol) for 20 min and washed twice with PBS. The cell treatments were examined under a light microscope (OPTIKA IM-3, OPTIKA, Italy) coupled to a digital camera (Optikam PRO8 Digital Camera C-P8, OPTIKA, Italy). Finally, the Oil Red O solution retained by the cells was extracted with 60% isopropanol, and the total lipid content was measured at 490 nm. Lipid content percentage (%) was calculated by dividing the absorbance of cells treated by the absorbance of the control (non-treated) cells and multiplied by 100.

#### 2.6.5 Cellular antioxidant activity

To evaluate the CAA of the *Crotalus* venom peptide-rich fractions, we used the pro-oxidant molecule AAPH as described by Gutiérrez-Grijalva et al. (2019). HepG2 cells were grown in DMEM supplemented with 10% FBS, and 1% Pen-Strep antibiotic and maintained at 37 °C in a humidified atmosphere of 5 % CO_2_. Cells were seeded in a 96-well plate (5 × 10^4^ cells/well) and allowed to adhere for 16–24 h. After that, cells were treated with 100 μL of *Crotalus* venom peptide-rich fractions (2 μg/mL) containing DCFH-DA (60 μM). Then, the cells were incubated for 20 min at 37 °C. After, the treatment solutions were discarded, and the cells were rinsed twice with PBS. Lastly, 100 μL of a 500 μM AAPH solution was added to each well, excluding the blank and negative controls. Cell blank control was incubated with DCFH-DA in absence of *Crotalus* venom peptide-rich fractions and AAPH, negative controls were incubated with *Crotalus* venom peptide-rich fractions in absence of AAPH, and positive control was incubated with AAPH in absence of *Crotalus* venom peptide-rich fractions. Fluorescence was measured at 485 nm (excitation) and 538 nm (emission) every 2 min for 90 min at 37 °C using a microplate reader. Equation (1) was utilized to calculate CAA values:

(1) $$CAA\;unit=1-\frac{\int SA\;}{\int CA}$$

Where ∫SA represents the integrated area under the curve of sample fluorescence versus time, and ∫CA represents the integrated area from the negative control curve.

#### 2.6.6 Nitric oxide determination

Nitric oxide production was measured in the macrophage cell line, Raw 264.7, according to Ortega-Hernández et al. (Ortega-Hernández et al., 2023). The Raw 264.7 cells were grown in DMEM supplemented with 5% FBS and 1% Pen-Strep antibiotic at 37 °C and 5% CO_2_. After reaching confluency, cells were seeded in a 96-well plate (5 × 10^4^ cells/well) and allowed to adhere for 16–24 h. To evaluate the effects of *Crotalus* venom peptide-rich fractions, 50 μL of each peptide-rich fraction (2 μg/mL) was added to the cells. After 4 h of incubation, half of the wells were stimulated with LPS at 1 μg/mL, while the remaining wells served as negative controls for each sample. The nitrite concentration in the cell culture supernatant was used to measure nitric oxide production. The amount of nitrite in the medium (100 μL) was measured with the Griess Reagent System (Promega, Madison, WI, USA) according to the manufacturer's directions. The absorbance readings were obtained at 550 nm on a Synergy HT plate reader (Bio-Tek Instruments, Inc., VT, USA) after 10 min incubation. A standard curve of sodium nitrite (1.5–50 μM) was prepared to quantify nitrate concentration. Untreated and LPS-stimulated cells were used as negative and positive controls, respectively. The percentage of nitric oxide inhibition (%) was calculated using Equation (2):

(2) $$Nitric\;oxide\;inhibition\;\left(\%\right)=\left(\frac{Sample\;-\;Nsc}{Pc-Nc}\right)\times 100$$

Where Sample represents the LPS-stimulated cells treated with *Crotalus* venom peptide-rich fractions, Nsc (Negative sample control) represents the LPS-free cells treated with *Crotalus* venom peptide-rich fractions, Pc (positive control) represents the LPS-stimulated cells, and Nc (Negative control) represents the LPS-free cells.

### 2.7 Data analysis

All experiments were performed by at least three independent replicates. The results were expressed as mean ± standard deviation. The experiments were analyzed by analysis of variance (ANOVA). When ANOVA showed significant differences (p < 0.05), the least significant difference (LSD) test was performed. LSD test allowed us to compare the mean of each venom sample with every other venom sample, identifying which venom samples have statistically different means. In the figures, the LSD statistical differences were denoted by letters above the bars, when two or more venom samples had a different letter, they did have statistical difference (p ≤ 0.05). The statistical analyses were done in Minitab 21 (PA, USA) and plotted in Prism Graph Pad 9.

## 3 Results

In this study, 54 samples of venom extracted from 18 *Crotalus* species and subspecies were used (Table 1). To test the therapeutic potential of the *Crotalus* venom peptide-rich fraction to treat type-2 diabetes and obesity, we used in vitro models to test insulin resistance and adipocyte lipid accumulation. Additionally, we evaluated the antioxidant and anti-inflammatory potential of the peptides due to obesity and type-2 diabetes can elicit oxidative stress and activate inflammatory pathways (Hurrle and Hsu, 2017; Panic et al., 2022).

### 3.1 Electrophoretic venom profile

**Fig. 1:**
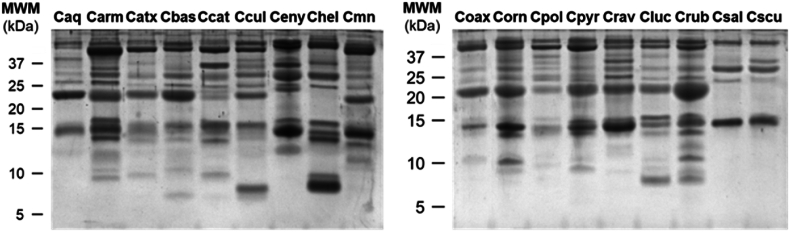
**Tricine-SDS-PAGE visualization of the toxin profile for the evaluated rattlesnake venom samples.** The venom samples (10 μg of each venom) were separated on a 16% Tricine-SDS-PAGE under reducing conditions and the gels were stained with Coomassie colloidal dye. Caq, *C. aquilus*; Carm, *C. armstrongi*; Catx, *C. atrox*; Cbas, *C. basiliscus*, Ccat: *C. catalinensis*; Ccul, *C. culminatus*; Ceny, *C. enyo*; Chel, *C. oreganus helleri*; Cmn, *C. molossus nigrescens*; Coax, *C. m. oaxacus*; Corn, *C. ornatus*; Cpol, *C. polystictus*; Cpyr, *C. pyrrhus*; Crav, *C. ravus*; Cluc, *C. ruber lucasensis*; Crub, *C. r. ruber*; Csal, *C. scutulatus salvini*; Cscu, *C. s. scutulatus*; MWM, molecular weight marker.

The whole venom profile (including proteins and peptides) from each tested species was observed through 16% Tricine-SDS-PAGE (Fig. 1). All the venom samples showed a complex pattern of proteins bands distributed from 6 to 50 kDa (Fig. 1), with a similar composition of the major and minor toxin bands: P-I (21–25 kDa) and P-III (45–55 kDa) snake venom metalloproteinases, PLA_2_ (12–15 kDa), snake venom serine proteases (30–40 kDa), C-type lectins (14–17 kDa), L-amino acid oxidases (>50 kDa), cysteine-rich secretory proteins (25–28 kDa) (Arnaud et al., 2021; Borja et al., 2018; Calvete et al., 2009; Durban et al., 2017; Franco-Servín et al., 2021; Lazcano-Pérez et al., 2022; Mackessy et al., 2018; Mackessy, 2010; Roldán-Padrón et al., 2022; Sánchez et al., 2020; Segura et al., 2017; Smith and Mackessy, 2016). In addition, several bands of low molecular weight (<10 kDa, hereafter referred to as peptides) were evident in the venom profiles.

### 3.2 Insulin resistance

**Fig. 2:**
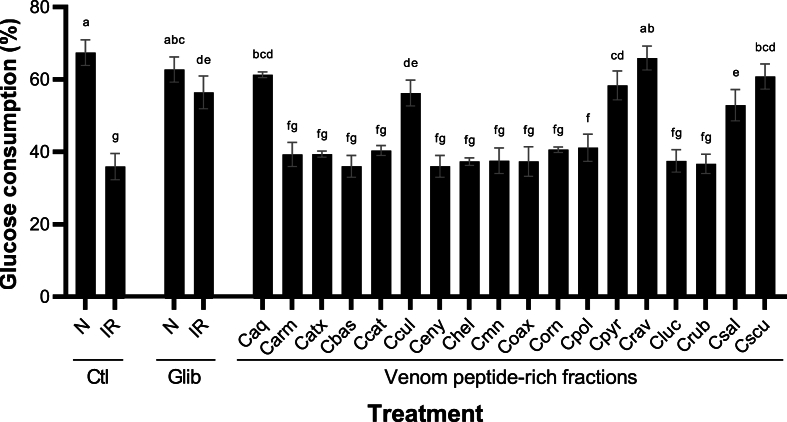
***In vitro* IR modulation by the *Crotalus* venom peptide-rich fractions in HepG2 cells.***Crotalus* venom peptide-rich fractions were tested using 5 μg/mL for 5 h. Negative control (N) was cultured in the absence of insulin, whereas IR control was cultured in the presence of insulin. Both controls were cultured in the absence (Ctl) and presence of 200 μM Glib. The results were expressed as mean (black boxes) ± standard deviation (gray bars). Letters above the bars indicate the statistical differences through LSD test, different letters denotated statistical differences between venom samples (p ≤ 0.05). Caq, *C. aquilus*; Carm, *C. armstrongi*; Catx, *C. atrox*; Cbas, *C. basiliscus*, Ccat: *C. catalinensis*; Ccul, *C. culminatus*; Ceny, *C. enyo*; Chel, *C. oreganus helleri*; Cmn, *C. molossus nigrescens*; Coax, *C. m. oaxacus*; Corn, *C. ornatus*; Cpol, *C. polystictus*; Cpyr, *C. pyrrhus*; Crav, *C. ravus*; Cluc, *C. ruber lucasensis*; Crub, *C. r. ruber*; Csal, *C. scutulatus salvini*; Cscu, *C. s. scutulatus*.

In the IR condition, the liver cells do not respond adequately to insulin signals, so they do not efficiently absorb glucose from the blood (Mather et al., 2013). As a result, blood glucose levels remain elevated. Our results demonstrated that the Crav peptide-rich fraction increased the glucose uptake (65.91 ± 3.30%) as high as the negative control (67.43 ± 3.52%) and the Glib IR-induced control (56.41 ± 4.53%) (Fig. 2). Other peptide-rich fractions such as Caq (61.30 ± 0.83%), Cscu (60.30 ± 3.48%), Cpyr (58.38 ± 3.99%), Ccul (56.24 ± 3.56%), and Csal (52.92 ± 4.33%) showed an increase in the glucose cellular uptake as high as the observed in Glib IR-induced control. On the other hand, the other peptide-rich samples did not have any glucose consumption modulation.

**Fig. 3:**
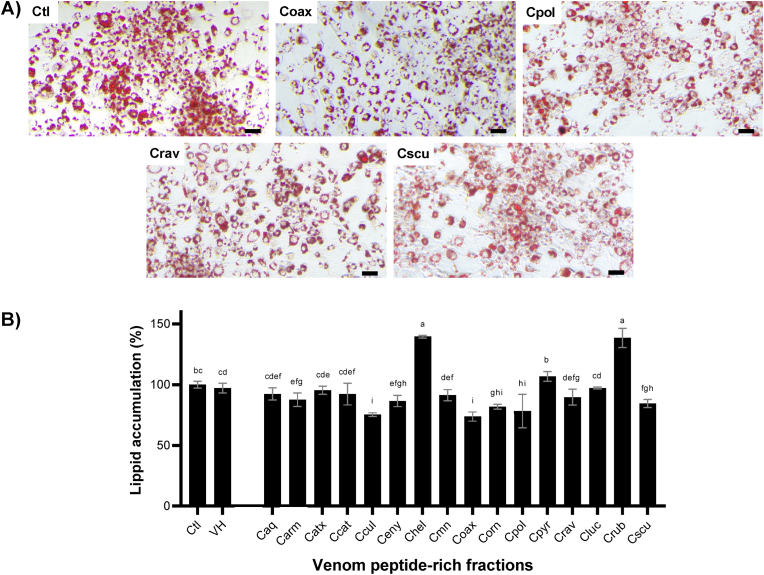
**Anti-obesogenic effect of the *Crotalus* venom peptide-rich fractions on white adipocyte differentiated 3T3-L1 cells.** A) Representative micrographs of 3T3-L1 cells treated with Crotalus venom peptide-rich fractions (Coax, Cpol, Crav, and Cscu) at the end of the differentiation process and stained with oil red O. These micrographs demonstrated the diminishment of lipid content. The micrographs were acquired with a 20X objective. Scale bar equal to 250 μm. B) The lipid content at the end of the experimentation was measured, through the extraction of the oil red O dye retained from 3T3-L1 cells. The results were expressed as mean (black boxes) ± standard deviation (gray bars). Letters above the bars indicate the statistical differences through LSD test, different letters denotated statistical differences between venom samples (p ≤ 0.05). Caq, *C. aquilus*; Carm, *C. armstrongi*; Catx, *C. atrox*; Cbas, *C. basiliscus*, Ccat: *C. catalinensis*; Ccul, *C. culminatus*; Ceny, *C. enyo*; Chel, *C. oreganus helleri*; Cmn, *C. molossus nigrescens*; Coax, *C. m. oaxacus*; Corn, *C. ornatus*; Cpol, *C. polystictus*; Cpyr, *C. pyrrhus*; Crav, *C. ravus*; Cluc, *C. ruber lucasensis*; Crub, *C. r. ruber*; Csal, *C. scutulatus salvini*; Cscu, *C. s. scutulatus*; Ctl, control; VH, vehicle. (For interpretation of the references to colour in this figure legend, the reader is referred to the Web version of this article.)

### 3.3 Lipid accumulation effect

To perform the lipid accumulation assay, we selected an experimental concentration of 25 μg/mL for all *Crotalus* venom peptide-rich fractions. This concentration generated a minimal effect on differentiated 3T3-L1 cell viability (85%) during 24 h exposure. The anti-obesogenic potential of the *Crotalus* venom peptide-rich fractions was evaluated during the differentiation process of 3T3-L1 cells using Oil Red O staining, this dye is used to stain neutral lipids, cholesteryl esters, and lipoproteins (Du et al., 2023). The effects of 25 μg/mL of peptide fractions on intracellular lipid accumulation are shown in Fig. 3, including micrographs of some of the fractions with the most significant effect (Fig. 3A). A reduction in lipid content was observed after treatment of 3T3-L1 cells with Coax, Cpol, Crav, and Cscu peptide fractions compared to the control. This change of morphology in droplet size has been associated with the browning of the adipose tissue (Dong et al., 2023).

The quantification of oil red O staining (Fig. 3B) demonstrated that Ccul, Coax, Cpol, and Cscu peptide fractions show the most significant diminishment of intracellular lipids accumulation (p < 0.05), reducing Oil Red O staining by 24.64 ± 1.56%, 26.24 ± 3.80%, 21.70 ± 13.97%, and 15.49 ± 3.37%, respectively, when compared to the control group. The micrographs and the quantification of the oil red O confirm that the peptide fraction of Coax venom has the most remarkable anti-obesogenic activity. On the other hand, Chel (139.50 ± 1.12%) and Crub (138.70 ± 7.86%) peptide-rich fractions incremented the quantity of lipids contained in differentiated 3T3-L1 cells.

### 3.4 Cellular antioxidant activity

**Fig. 4:**
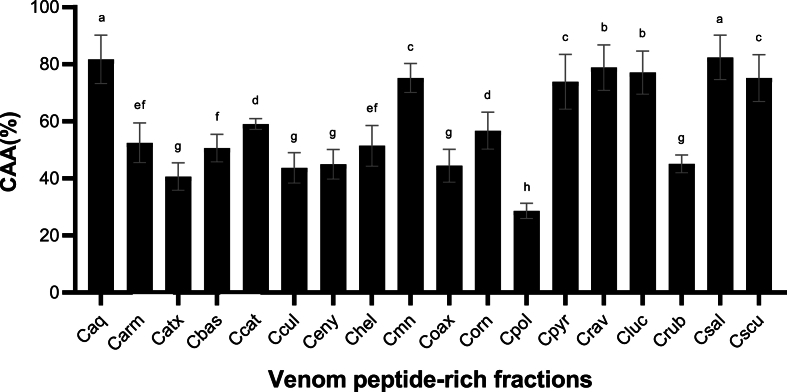
**Cellular antioxidant activity (CAA) percentage of the *Crotalus* venom peptide-rich fractions on HepG2 cells.** The antioxidant activity was evaluated incubating the HepG2 cells with the Crotalus venom peptide-rich fractions (2 μg/mL) to prevent the pro-oxidant effect of AAPH. The results were expressed as mean (black boxes) ± standard deviation (gray bars). Letters above the bars indicate the statistical differences through LSD test, different letters denotated statistical differences between venom samples (p ≤ 0.05). Caq, *C. aquilus*; Carm, *C. armstrongi*; Catx, *C. atrox*; Cbas, *C. basiliscus*, Ccat: *C. catalinensis*; Ccul, *C. culminatus*; Ceny, *C. enyo*; Chel, *C. oreganus helleri*; Cmn, *C. molossus nigrescens*; Coax, *C. m. oaxacus*; Corn, *C. ornatus*; Cpol, *C. polystictus*; Cpyr, *C. pyrrhus*; Crav, *C. ravus*; Cluc, *C. ruber lucasensis*; Crub, *C. r. ruber*; Csal, *C. scutulatus salvini*; Cscu, *C. s. scutulatus*.

The CAA measured the ability of the venom peptide-rich fractions to react or neutralize the reactive oxygen species (ROS) generated in the cells after an oxidant stimulus elicited by AAPH. According to our results, the venom peptide-rich fractions with the higher antioxidant potential were Csal (82.41 ± 7.82%), Caq (81.70 ± 8.52%), Crav (78.86 ± 7.95%), and Cluc (77.09 ± 7.54%), inhibiting about 80% of AAPH ROS production (Fig. 4). In contrast, less antioxidant activity was shown by Cpol (28.65 ± 2.66%) peptide-rich fraction.

### 3.5 Nitric oxide production inhibition

**Fig. 5:**
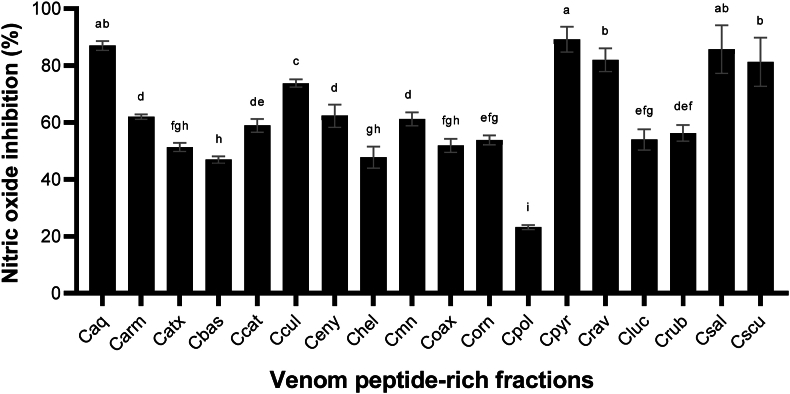
**Nitric oxide inhibition by *Crotalus* venom peptide-rich fractions on Raw 264.7 cells.** To evaluate the nitric oxide production, the Raw 264.7 cells were incubated with the Crotalus venom peptide-rich fractions (2 μg/mL). After the peptide-rich fraction incubation, nitric oxide production was stimulated with LPS (1 μg/mL). The results were expressed as mean (black boxes) ± standard deviation (gray bars). Letters above the bars indicate the statistical differences through LSD test, different letters denotated statistical differences between venom samples (p ≤ 0.05). Untreated and LPS cells were used as positive control. Caq, *C. aquilus*; Carm, *C. armstrongi*; Catx, *C. atrox*; Cbas, *C. basiliscus*, Ccat: *C. catalinensis*; Ccul, *C. culminatus*; Ceny, *C. enyo*; Chel, *C. oreganus helleri*; Cmn, *C. molossus nigrescens*; Coax, *C. m. oaxacus*; Corn, *C. ornatus*; Cpol, *C. polystictus*; Cpyr, *C. pyrrhus*; Crav, *C. ravus*; Cluc, *C. ruber lucasensis*; Crub, *C. r. ruber*; Csal, *C. scutulatus salvini*; Cscu, *C. s. scutulatus*.

Nitric oxide is an important biomarker involved in different physiological processes. Its synthesis is generated by the nitric oxide synthase, and can be elicited due to proinflammatory agents, including LPS, to mediate the host innate immune response to pathogens (Yoon et al., 2009; Yoshikawa et al., 2000). Typically, nitric oxide (NO) production plays a vital role in combating bacteria and viruses. However, excessive NO production can lead to conditions such as sepsis or inflammatory diseases, including asthma, rhinitis, and cancer (Hong et al., 2023). Regulation of nitric oxide production is desirable for inti-inflammatory molecules. Therefore, we evaluated the potential anti-inflammatory activity of the venom peptide fractions through nitric oxide production modulation. Our results showed an important nitric oxide production reduction of 89.22, 85.72, and 87.02% elicited by Cpyr, Caq, and Csal venom peptide-rich fractions, respectively (Fig. 5). Cpol peptide-rich fraction showed the lower anti-inflammatory potential, exhibiting only 23% inhibition of nitric oxide.

## 4 Discussions

### 4.1 Electrophoretic venom profile

The peptide bands in *Crotalus* venom samples were evident in almost all venom samples except Caq, Ceny, Csal, and Cscu (Fig. 1). Nevertheless, even when the peptide content was not visible in the electrophoretic analysis, the peptide quantification method used in this research (Pierce Quantitative Colorimetric Peptide Assay Kit) demonstrated the presence of the peptides. These bands were observed in the molecular weight range of the Dis (7–12 kDa) and Ctm-like peptides/myotoxins (6–8 kDa). The venoms of Carm, Catx, Cbas, Ccat, Ccul, Chel, Coax, Corn, Cpol, Cpyr, Crav, Cluc, and Crub showed bands in the reported molecular weight of Dis; interestingly, only Catx, Ccat, and Cpol venom had been described for having this toxin family in its venom (Arnaud et al., 2021; Calvete et al., 2009; Mackessy et al., 2018). On the other hand, the samples of Carm, Cbas, Ccat, Ccul, Chel, Corn, Cluc, and Crub showed bands with a molecular mass similar to Ctm-like myotoxin peptides. However, the presence of this toxin family has been described only for Cbas, Ccul, and Chel venoms (Ponce-López et al., 2021). Furthermore, other toxin families that are present in *Crotalus* venoms, such as natriuretic peptides, bradykinin-potentiating peptides and tripeptide inhibitors (Mackessy, 2010). Nevertheless, our results did not show bands with a similar molecular mass to these toxin families.

### 4.2 Insulin resistance

Some isoforms of the PLA_2_, Dis, and Ctm-like myotoxin families have been described to show modulatory effect in insulin resistance models (Marinovic et al., 2018; Moore et al., 2015; Nogueira et al., 2005; Toyama et al., 2005), as Glib, these peptides can sensitize the β-pancreatic cells to insulin (Finneran and Landon, 2018). The Ccul peptide-rich fraction was the only one among the different rattlesnake venoms that improved the glucose uptake in the IR model, from which a proteomic characterization has confirmed the presence of Dis and Ctm-like peptides (Durban et al., 2017). Besides, the Cpyr and Crav venoms had toxins with an apparent molecular mass similar to Dis and Ctm-like peptides, whereas, in the Caq, Csal, and Cscu venom samples, bands with this molecular weight were not observed (Fig. 1).

The mechanism of action of these toxins to generate the modulation of IR has yet to be fully understood. Nevertheless, Ctm-like peptides are assumed to induce this effect by modulating of the Nav channels present in β-pancreatic cells (Toyama et al., 2005; Toyama, 2000). Moreover, it has been demonstrated that margatoxin, isolated from the Bark Scorpion (*Centruroides margaritatus*) venom, which shares a similar molecular mechanism with Ctm-like peptides, increases the glucose uptake through the inhibition of Kv1.3 channels (Li et al., 2006). On the other hand, Dis may play a role in activating the β-pancreatic cell signaling pathways through receptor tyrosine kinases (Moore et al., 2015). These results suggest that Crav, Caq, Cscu, Cpyr, Ccul, and Csal peptide-rich fractions contain peptides that may serve to mitigate IR on type-2 diabetic patients.

### 4.3 Lipid accumulation effect

The adipocyte reduction of lipid droplet size and lipid content generated by Ccul, Coax, Cpol, and Cscu peptide-rich fractions (Fig. 3) suggest that the peptides contained in these fractions activate lipid catabolism and may generate browning on adipocytes. This phenomenon is especially important, as the fat-burning generated by brown adipocyte cells is related to body weight loss and IR mitigation (Cheng et al., 2021). This phenomenon is only described for two snake venom toxins, Ctm from *C. d. terrificus* and pOh2 from the king cobra (*Ophiophagus hannah*). Both toxins reduced lipid accumulation in adipocytes (Marinovic et al., 2018; Nguyen et al., 2017).

The mechanism of action of both toxins to elicit the lipid accumulation diminishment remains unclear. Nevertheless, the mechanism of action of these peptide fractions to promote this reduction of lipid accumulation could be induced through Kv1.3 channel modulation since this channel regulates energy homeostasis and body weight (Xu, 2003). It has been reported that venom toxins such as ShK-186 and margatoxin can inhibit the Kv1.3 channel to induce insulin sensitivity and diminish lipid accumulation (Li et al., 2006; Upadhyay et al., 2013). Moreover, a similar effect was observed in mice treated with a Kv channel modulator Ctm from *C. d. terrificus* venom (Peigneur et al., 2012), generating browning in adipocytes (Marinovic et al., 2018). Other Ctm-like peptides have been described or visualized in Ccul, Cpol, and Cscu venoms, which can explain the results observed in 3T3-L1 cells (Borja et al., 2014; Mackessy et al., 2018; Ponce-López et al., 2021).

On the other hand, Chel and Crub peptide-rich fractions increased the lipid content on 3T3-L1 (Fig. 3). This suggests that these peptide-rich fractions are still inducing adipocyte differentiation. Nevertheless, to ensure that the Chel and Crub peptide-rich fractions are inducing adipocyte differentiation, the activation of other markers such as the peroxisome proliferator-activated receptor γ and CCAAT/enhancer-binding protein α must be measured (Choi and Yoo, 2018).

### 4.4 Cellular antioxidant activity

Snake venoms and their toxins, such as snake venom metalloproteinases, PLA_2_, and L-amino acid oxidases, are traditionally recognized for their pro-oxidant activity during envenomation (dos Reis et al., 2022; Sunitha et al., 2015), while no antioxidant activity has been previously reported in the *Crotalus* venoms. The BmT-2 toxin isolated from the Brazilian Lancehead (*Bothrops moojeni*) venom and salamandrin-I from the Fire Salamander (*Salamandra*) skin secretions was demonstrated to generate antioxidant activity (Dematei et al., 2022; Plácido et al., 2020). Both toxins are aromatic-rich peptides, in which Trp and Tyr residues could function as an electron acceptor when exposed to AAPH (Fuentes-Lemus et al., 2016; Zheng et al., 2016), suggesting that small peptides rich in aromatic residues from snake venoms can act as an antioxidant component. The presence of the two Trp and one Tyr residues in the Ctm-like peptides surface (Coronado et al., 2013) could be responsible for the antioxidant activity found in this work. However, a more detailed analysis must be performed to corroborate this hypothesis.

The antioxidant effect observed in Csal, Caq, Crav, and Cluc peptide-rich fractions may be beneficial during type-2 diabetes and obesity treatments. Both diseases increase oxidant stress, exacerbating its long-term effects. Here, the *Crotalus* venom peptides may have a dual effect, acting as anti-diabetic and anti-obesogenic drugs and mitigating oxidative stress.

### 4.5 Nitric oxide production inhibition

Several snake venoms and isolated toxins are reported to modulate macrophage metabolism and functioning (Sampaio et al., 2001), including Ctm (Lee et al., 2016), crotoxin (Neves et al., 2023; Sampaio et al., 2003), PLA2 (Zuliani et al., 2024). Particularly, the increase in nitric oxide production is described for the whole venom of the Golden Lancehead (*B. insularis*) venom (Alberto-Silva et al., 2020), as well as the peptide fraction of the Yarará Lancehead (*B. jararaca*) venom (Menezes et al., 2016) and pure Ctm from *C. d. terrificus* venom (Lee et al., 2016). In contrast, an RGD-containing Dis isolated from the Brown spotted pit viper (*Protobothrops mucrosquamatus*) venom is capable of down-regulate the expression of the inducible nitric oxide synthase on Raw 264.7 cells (Hung et al., 2016). This phenomenon is elicited through Dis interaction with αVβ3 integrin, blocking MAP kinase and activating of NF-κB transcription factor. *Crotalus* venoms are rich sources of Dis toxins (Sánchez et al., 2006; Scarborough et al., 1993; Soto et al., 2007), suggesting that they could be involved in the inhibition of inflammatory response by a similar mechanism reported for *P. mucrosquamatus*.

The suppression of the inflammatory process generated by Cpyr, Caq, and Csal venom peptide-rich fractions may be relevant during type-2 diabetes and obesity treatment. Both diseases are associated with an excessive inflammatory response. Therefore, anti-inflammatory activity present in Crotalus peptides may be beneficial during type-2 diabetes and obesity treatment.

This research demonstrated that the peptides contained in peptide-rich fractions from Caq, Cpyr, Crav, Csal, and Csu are relevant to revert IR, diminishing lipid accumulation, and mitigate oxidative stress and inflammation. Nevertheless, to develop a drug to treat any of these health problems several steps must be followed. First, activity-guided isolation must be performed to have an active isolated peptide. Then, corroborate the in vitro and in vivo activity of the peptide. And finally, test the effectiveness and safety of the peptide in preclinical models.

## 5 Conclusions

This work showed that the peptide-rich fractions from *Crotalus* venoms contain peptides that can revert IR, diminish lipid accumulation, and mitigate oxidative stress and inflammation on in vitro models. Notably, Caq, Cpyr, Crav, Csal, and Cscu peptide-rich fractions demonstrated significant potential to revert the IR condition and mitigate oxidative stress. Ccul peptide-rich fraction was able to modulate IR and lipid accumulation. Coax, Corn, and Cpol peptide-rich fractions only modulated lipid accumulation. Finally, Cmn and Cluc peptide-rich fractions were able to reverse cellular oxidation. These results demonstrated the therapeutic potential of the peptides contained in the *Crotalus* venoms to develop novel toxin-inspired drugs to treat metabolic diseases such as type-2 diabetes and obesity.

## Funding

This research was funded by the Challenge-Based Research Funding Program 2022 grants: I002-IOR002-C6-T2-E, I018-IOR001-C5-T1-E and IOR001-C5-T1 from Tecnologico de Monterrey, and Ciencia de Frontera 2023 grant (CF-2023-I-2019) from the Mexican National Council for Science and Technology.

## Ethical statement

Venom collection was performed under permission of the Dirección General de Vida Silvestre of Secretaría de Medio Ambiente y Recursos Naturales de México (ACS: SGPA/DGVS/011587/17, GAF: SPARN/DGVS/04499/23, AGC: SGPA/DGVS/7609/19-DGVS/03663).

## CRediT authorship contribution statement

**David Meléndez-Martínez:** Writing – review & editing, Writing – original draft, Visualization, Methodology, Investigation, Formal analysis, Conceptualization. **Erika Ortega-Hernández:** Visualization, Methodology, Investigation, Formal analysis. **Edwin Estefan Reza-Zaldívar:** Visualization, Methodology, Investigation, Formal analysis. **Alejandro Carbajal-Saucedo:** Writing – review & editing, Resources, Investigation. **Gustavo Arnaud-Franco:** Writing – review & editing, Resources, Investigation. **Ana Gatica-Colima:** Writing – review & editing, Resources, Investigation. **Luis Fernando Plenge-Tellechea:** Writing – review & editing, Resources, Investigation. **Marilena Antunes-Ricardo:** Writing – review & editing, Writing – original draft, Supervision, Methodology, Funding acquisition, Formal analysis, Conceptualization. **Daniel A. Jacobo-Velázquez:** Writing – review & editing, Visualization, Supervision, Funding acquisition, Formal analysis, Conceptualization. **Karla Mayolo-Deloisa:** Writing – review & editing, Visualization, Supervision, Resources, Project administration. **Omar Lozano:** Writing – review & editing, Supervision, Project administration. **Marco Rito-Palomares:** Writing – review & editing, Supervision, Funding acquisition. **Jorge Benavides:** Writing – review & editing, Writing – original draft, Supervision, Project administration, Methodology, Funding acquisition, Formal analysis, Conceptualization.

## Declaration of competing interest

The authors declare that they have no known competing financial interests or personal relationships that could have appeared to influence the work reported in this paper.

Our research work has received financial support from CONACyT (Mexican 10.13039/501100003141National Council of Humanities, Sciences and Technologies), as part of the “Ciencia de Frontera 2023” grant (CF-2023-I-2019). CONACyT has not participated in any way in the definition, execution, and validation of the results of the project. CONACyT, as a governmental agency, focuses on promoting scientific and technological development in Mexico, having no commercial activity. Therefore, CONACyT holds no commercial interest in the results of the project, nor has any influence on such results. In this context, this external funding does not represent a competing financial interest.

## Data Availability

Data will be made available on request.
